# MicroRNAs miR-26a, miR-26b, and miR-29b accelerate osteogenic differentiation of unrestricted somatic stem cells from human cord blood

**DOI:** 10.1186/1471-2164-14-111

**Published:** 2013-02-19

**Authors:** Hans-Ingo Trompeter, Janine Dreesen, Eugenie Hermann, Katharina M Iwaniuk, Markus Hafner, Neil Renwick, Thomas Tuschl, Peter Wernet

**Affiliations:** 1 University Düsseldorf, Medical Faculty, Institute for Transplantation Diagnostics and Cell Therapeutics (ITZ), Düsseldorf, D-40225, Germany; 2 Howard Hughes Medical Institute, Laboratory of RNA Molecular Biology, Rockefeller University, New York, NY, 10065, USA

**Keywords:** Cord blood stem cells, Osteogenic differentiation, MicroRNA expression, MicroRNA function, MicroRNA target identification

## Abstract

**Background:**

MicroRNAs are a population of short non-coding RNAs with widespread negative regulatory impact on mRNA translation. Unrestricted somatic stem cells (USSC) are a rare population in human cord blood that can be induced into cells representative of all three germinal layers. Here we analyzed the functional impact of miRNAs on the osteogenic differentiation in USSC.

**Results:**

Gene expression profiling identified 20 microRNAs that were consistently upregulated during osteogenic differentiation of two different USSC cell lines (SA5/73 and SA8/25). Bioinformatic target gene prediction indicated that among these microRNAs, miR-10a, -22, -26a, -26b, and -29b recognize transcripts that encode a set of proteins inhibiting osteogenesis. We subsequently verified osteo-inhibitory CDK6, CTNNBIP1, HDAC4, and TOB1 and osteo-promoting SMAD1 as targets of these microRNAs. In Western blot analyses demonstrated that endogenous levels of CDK6 and HDAC4 were downregulated during osteogenic differentiation of USSC and reduced following ectopic expression of miR-26a/b and miR-29b. In contrast, endogenous expression of SMAD1, targeted by miR-26a/b, was unaltered during osteogenic differentiation of USSC or following ectopic expression of miR-26a/b. Functional overexpression analyses using microRNA mimics revealed that miR-26a/b, as well as miR-29b strongly accelerated osteogenic differentiation of USSC as assessed by Alizarin-Red staining and calcium-release assays.

**Conclusions:**

miR-26a/b and miR-29b are upregulated during osteogenic differentiation of USSC and share target genes inhibiting osteogenesis. Furthermore, these microRNAs accelerate osteogenic differentiation, likely mediated by osteo-inhibitory proteins such as CDK6 and HDAC4.

## Background

microRNA (miRNA)-mediated translational repression is an important regulatory mechanism in multiple cellular processes. miRNAs are a subpopulation of small RNAs, averaging 22 nucleotides in length, which inhibit translation through sequence-specific binding to target sites within the 3^′^-UTRs of mRNAs. Following transcription, miRNAs are processed in a two-step mechanism involving the RNAses DROSHA and DICER [1,2] and subsequently integrated into the RNA-induced silencing complex [3,4], thereby unfolding their regulatory potential to regulate mRNAs [5-7]. Exhibiting stage- and tissue-specific expression patterns during development [8,9], miRNAs not only function as key regulatory molecules in multiple cellular processes including apoptosis [10,11], cancer [12], proliferation [13], development [14], and differentiation [15] but also control stemness and pluripotency of embryonic stem cells e.g. by repressing pluripotency factors OCT4, SOX2 and KLF4 [16].

Osteogenesis is a highly coordinated process involving transcription factors, such as RUNX2 and OSTERIX [17,18], BMP2, and other factors, that drive committed stem cells toward fully differentiated osteocytes (reviewed in [19-21]). Osteogenesis is promoted through several signalling pathways, including WNT/ß-catenin, BMP, JAK/STAT, and MAPK [22-27]. Several miRNAs modulate osteogenic differentiation: miR-125b negatively regulates osteoblastic differentiation through targeting VDR, ErbB2, and Osterix [28,29]; miR-133 (targeting RUNX2) and miR-135 (recognizing SMAD5) inhibit differentiation of mouse osteoprogenitors [30]; miR-26a and miR-29b facilitate osteogenic differentiation of human adipose tissue-derived stem cells (hADSCs), and positively modulate mouse osteoblast differentiation [31,32]. Multiple other miRNAs, including miR-9, -17, -27, -30, -96, -106, -138, -181, -182, -320, and −326, have been linked to osteogenesis [30].

Unrestricted somatic stem cells (USSC) are a rare CD45-negative population in human cord blood [33]. These cells can be discriminated from CB-MSC and BM-MSC by their HOX expression pattern which resembles that of H9 embryonic stem cells [34]. Adherently-growing *in vitro* USSC can be induced to cells representative of all three germinal layers on a clonal level [35] and have been successfully reprogrammed to a pluripotent ES-like state [36]. Undergoing miRNA-supported cell cycle arrest, USSC can be differentiated into cells of neural lineage with miRNAs acting as network-like regulators [37-39]. USSC also differentiate into functional hepatic-like cells [40,41] as well as along osteogenic and chondrogenic lineages [33]. Upon induction with dexamethasone, ascorbic acid, and ß-glycerol phosphate (DAG), USSC differentiate into osteoblasts as evidenced by calcium phosphate deposition, bone-specific ALP-activity, increase in Ca^2+^-release, and expression of the osteogenic marker proteins osteocalcin, osteopontin, bone sialo-protein, and collagen type I [33]. Bony reconstitution was observed following implantation of USSC into nude rat femurs [33]. Beside their differentiation potential, USSC also fulfil regenerative functions in acute spinal cord trauma [42].

Here we analyzed the impact of miRNAs on osteogenic differentiation of USSC. We identified a set of miRNAs upregulated upon induction of osteogenesis, co-ordinately regulating a distinct set of genes known to inhibit osteogenesis. Among these inhibitors, CDK6, CTNNBIP1, HDAC4, TGFB3, and TOB1 were experimentally identified as targets of miR-26a, miR-26b, and miR-29b. These miRNAs were functionally identified as accelerators of osteogenic differentiation of USSC.

## Results

### Differential miRNA expression during osteogenic differentiation of USSC

**Figure 1:**
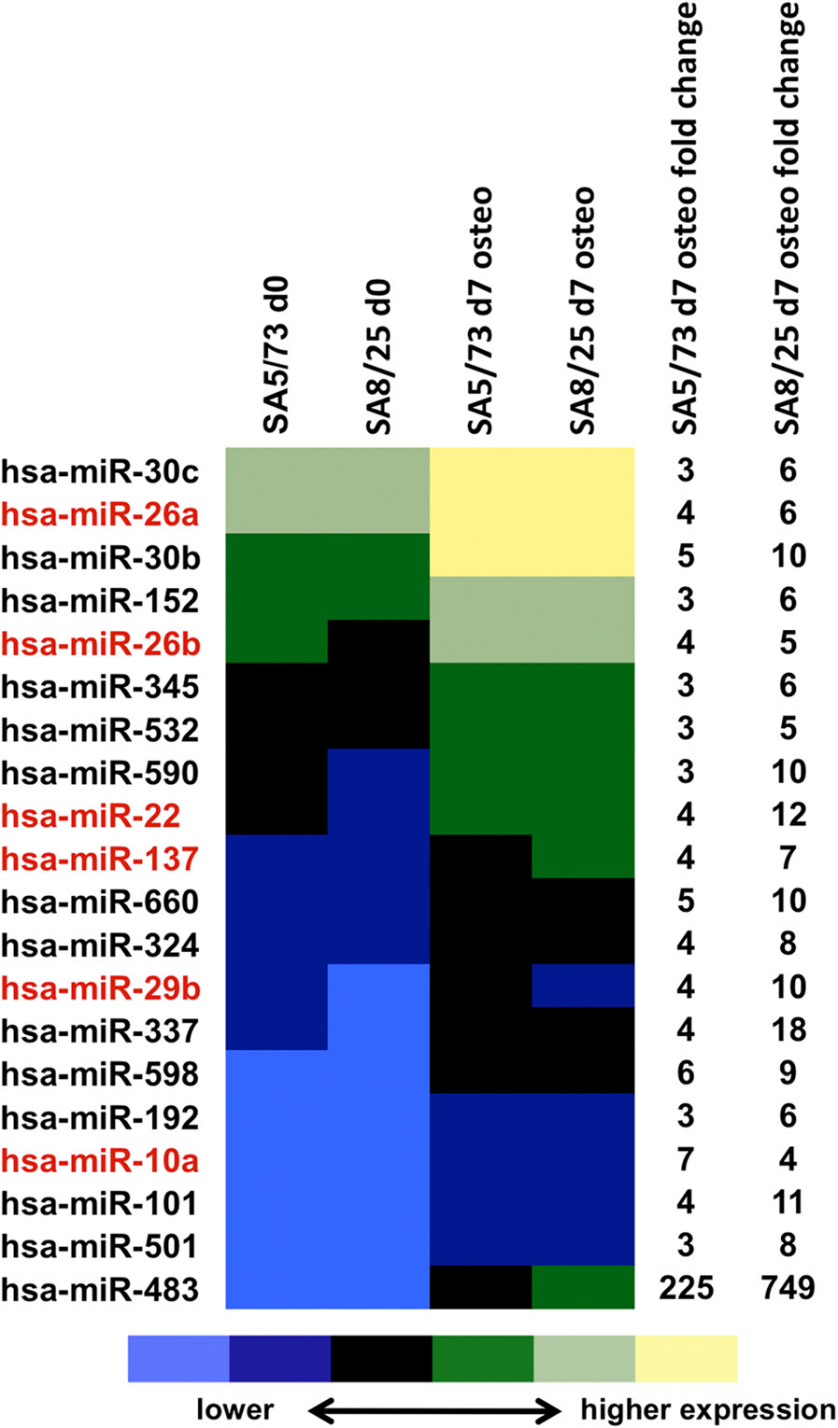
**Differential miRNA expression during DAG-mediated osteogenic differentiation of USSC lines SA5/73 and SA8/25.** The heat map shows common upregulation of 20 miRNAs in both cell lines as measured by qRT-PCR, together with the fold changes (2^–ddCt^) of miRNA expression in DAG-induced USSC at day 7 of osteogenic differentiation compared to the native cells. MicroRNAs highlighted in red were selected for further studies. Full miRNA expression data are presented in Additional file 1.

To assess the impact of miRNAs on osteogenic differentiation of USSC we studied two USSC lines (USSC SA5/73 and USSC SA8/25) that were induced to osteogenic differentiation using DAG as described [33]. As strong calcification of USSC during osteogenic differentiation impacts RNA isolation, we restricted our analyses to day 7 of differentiation. miRNA expression profiles of native and day 7 osteo-differentiated USSC were analyzed using the RT-PCR-based TaqMan Assay (Pool A) covering 377 miRNAs [43]. In SA5/73, 220 miRNAs were expressed and 124 miRNAs were upregulated by a factor ≧ 2 in differentiated cells. In SA8/25, 225 miRNAs were expressed and 196 miRNAs were upregulated during osteogenic differentiation. Interestingly, only 30 miRNAs were commonly upregulated in both USSC lines. In follow-up analyses we focused on 20 of these microRNAs (Figure 1), which were not only upregulated by a factor ≧ 2 but also present at high expression levels (< Ct 26) in differentiated USSC. We omitted those upregulated miRNAs that were weakly expressed in differentiated USSC due to their expected minor biological impact. Among the most prominently expressed miRNAs were miR-10a, miR-152, miR-22, miR-26a/b, miR-29b, miR-30b/c, miR-345, and miR-532-5p. Complete miRNA expression data from USSC SA5/73 and SA8/25 osteogenic differentiation experiments are presented in Additional file 1.

### Bioinformatic target gene predictions

To investigate the biological impact of our selected set of 20 miRNAs, we computationally identified miRNA targets using the DIANA miRGen target gene prediction software [44], which combines the prediction results of several web-based algorithms (TargetScan, PicTar-4, PicTar-5, miRanda, DIANA microT). This approach resulted in an extensive list of putative targets (>10^4^ genes), many of which were recognized by more than one of our 20 miRNAs. As our study is focused on miRNAs that are upregulated during osteogenic differentiation, we reasoned that their biological impact should be posttranscriptional downregulation of proteins inhibiting osteogenic differentiation. We thus filtered our list of putative targets using the *Gene Ontology* (GO) terms function implemented in the DAVID database, applying search terms like “bone” and “osteo”. Table 1 presents an overview of 21 proteins inhibiting osteogenic differentiation that are putatively recognized by our set of 20 miRNAs. Consistent with the view that miRNAs regulate networks [38], Table 1 demonstrates target gene redundancy: certain proteins were predicted to be targeted by more than one miRNA and other miRNAs (e.g. miR-29b) putatively regulate up to 11 proteins (Table 1).

**Table 1:** Bioinformatic target predictions for the subset of 20 microRNAs upregulated in osteogenic differentiation of USSC

											**hsa-miR-**										
**Protein**	**Total**	**101**	**10a**	**137**	**152**	**192**	**22**	**26a**	**26b**	**29b**	**30b**	**30c**	**324-**	**337**	**345**	**483**	**501**	**532**	**590**	**598**	**660**
													**3p**								
**CDK6**	**6**	**x**		**x**			**x**	**x**	**x**	**x**											
**CTNNBIP1**	**5**		**x**			**x**		**x**	**x**	x*											
AHSG	**4**									**x**			**x**					**x**			**x**
**HDAC4**	**4**		**x**				**x**			x*					**x**						
CHRD	**3**						**x**							**x**		**x**					
COL1A1	**3**							**x**	**x**	**x**											
SOX2	**3**							**x**	**x**										**x**		
COL4A2	**2**							**x**		x*											
**DUSP2**	**2**									x*											**x**
HIPK2	**2**							**x**	**x**												
NOG	**2**				**x**	**x**															
SKI	**2**									**x**									**x**		
SMAD7	**2**	**x**					**x**														
SOSTDC1	**2**							**x**	**x**												
STATH	**2**	**x**		**x**																	
**TGFB3**	**2**					**x**				x*											
**TOB1**	**2**							**x**	**x**												
ACVR2A	**1**					**x**															
AREG	**1**														**x**						
COL5A3	**1**									**x**											
**SMAD6**	**1**									**x**											

Bioinformatic target predictions for the subset of 20 miRNAs upregulated in osteogenic differentiation of USSC on osteo-inhibitory proteins: miR-26a, -26b, and -29b were experimentally assessed for impact on proteins denoted in bold. The second column (“Total”) illustrates the number of miRNAs from the analyzed set that targets an individual protein. Predictions with an asterix were already validated in the literature.

Computational predictions indicated that the BMP-2 antagonist CDK6 [45-47] was recognized by six miRNAs (miRs-101, -137, -22, -26a, -26b, and -29b); CTNNBIP1 (also termed ICAT), an inhibitor of ß-catenin mediated transcription [48], was likely regulated by five miRNAs (miRs-10a, -192, -26a, -26b, and -29b); and Runx2 co-repressor HDAC4 [49] was a putative target of four miRNAs (miRs-10a, -22, -29b, and −345). Additional putative osteogenesis-inhibiting target proteins included DUSP2 [50], TOB1 [51], TGFB3 [52,53], and SMAD6 [54] (see Table 1 for target predictions). Interestingly, miR-26a and miR-26b were also predicted to regulate SMAD1, a positive regulator of osteogenic differentiation [55].

The most redundant miRNA-target network involved miR-26a/b and miR-29b and, to a lesser extent, miR-22, miR-10a, and miR-137 (Table 1); subsequent analyses focused on these six miRNAs. To avoid errors in measuring the expression of highly homologous miR-26a and -26b, we verified our TaqMan miRNA expression data from USSC SA5/73 and SA8/25 (Figure 1) in native USSC lines SA5/73, SA8/25, SA8/77 and SA4/101 and in (day 7) osteo differentiated SA8/77 and SA4/101, using an established small RNA sequencing method [56,57]. miR-26a and miR-26b expression was confirmed in all native USSC lines and both miRNAs were upregulated in differentiated SA8/77 and SA4/101 lines (Additional file 1).

### Experimental validation of predicted miRNA targets

Next, we experimentally verified miRNA regulation of selected targets (Table 1) using an established target validation assay [38,39]. Among these putative targets, CDK6, CTNNBIPI, TOB1, and HDAC4 contained the most predicted miRNA binding sites, whereas DUSP2, TGFB3, and SMAD6 each contained a solitary putative miR-29b target site. PCR amplification products representing the 3^′^-UTR of target genes were cloned at the 3^′^-end of the *firefly*-ORF of dual luciferase (*firefly* and *renilla*) vector pmirGLO. Based on the patterns of predicted miRNA binding sites on individual 3^′^-UTRs (see Additional file 2 for details), we devised the following cloning strategy for target validation: CDK6 3^′^-UTR (10235 bp) was cloned as two subfragments ranging from bases 147–4275 (CDK6-1) and 5825–9997 (CDK6-2); CTNNBIP1 (2442 bp) and TGFB3 (1088 bp) 3^′^-UTRs were covered by 1094bp and 277 bp fragments respectively. The HDAC4 3^′^-UTR (4933 bp) was represented by a 964 bp PCR-fragment (HDAC4-1) and two additional 65 bp and 57 bp double-stranded oligonucleotides (HDAC4-O1, -O2) covering single miRNA binding sites. Using oligonucleotides instead of longer 3^′^-UTR fragments is well described in the literature (e.g. see [58]). TOB1, DUSP2, SMAD1, and SMAD6 3^′^UTRs were respectively represented by double-stranded oligonucleotides of 65 bp, 58 bp, 37 bp, and 116 bp. In addition, an oligonucleotide pair with mutated seed sequences was used for TOB1.

**Figure 2:**
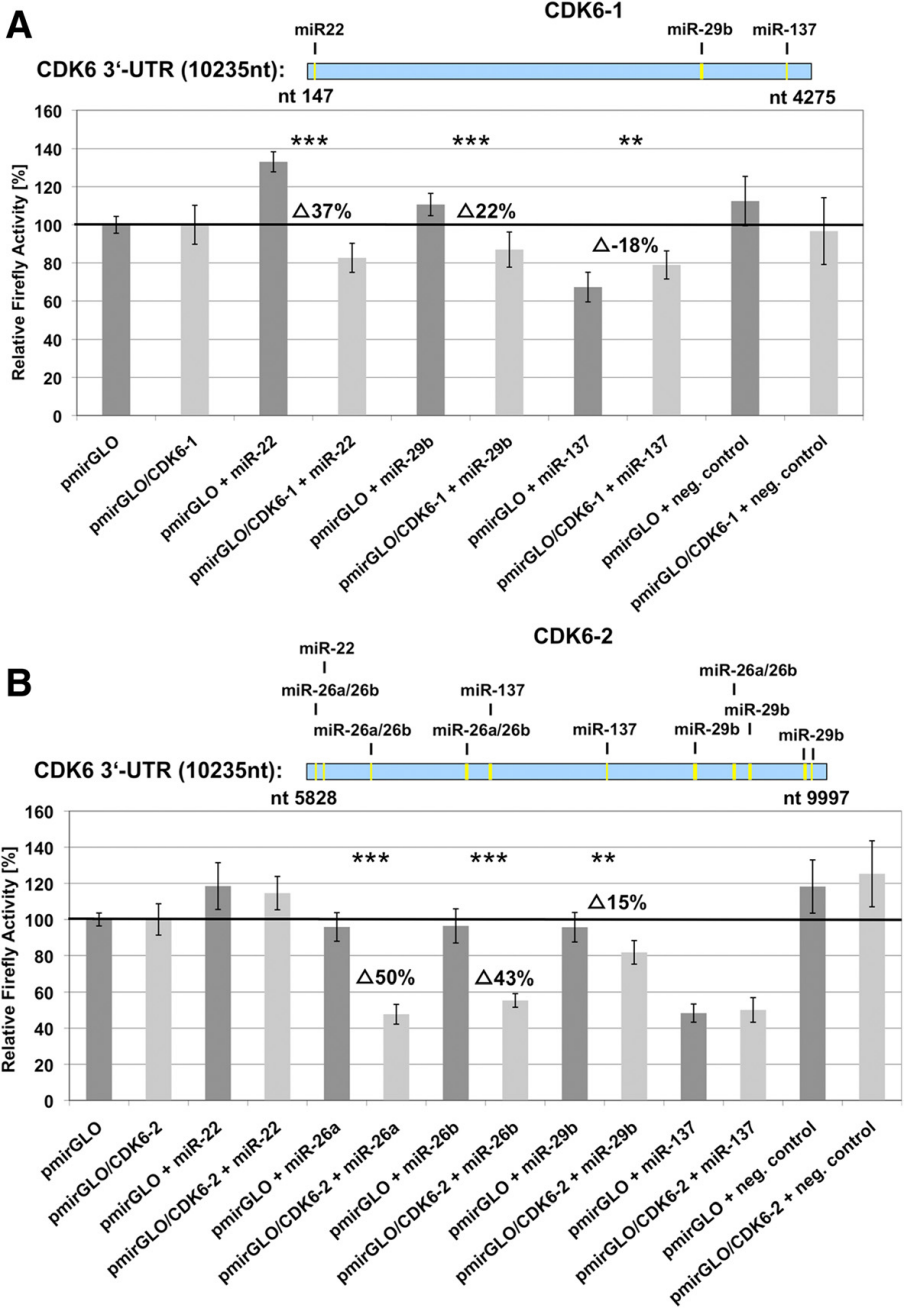
**Experimental validation of target gene predictions.** Experimental validation of putative target sites on 4128bp (**A**) and 4169bp (**B**) fragments of the 10235nt 3^′^-UTR of CDK6 (CDK6-1 and CDK6-2, shown as blue bars with indicated putative miRNA binding sites) for miRNAs miR-22, -26a, -26b, -29b, and -137 in HEK293T-Cells. To assess the influence of endogenous miRNAs, empty *firefly/renilla* dual reporter vector pmirGLO and pmirGLO/CDK6-3^′^-UTRs were each transfected into HEK293T-cells. Normalized *firefly*-activities were compared to those of pairwise co-transfections of these vectors with the miRNA mimic of interest (including an unspecific siRNA negative control) to test for unspecific effects of the given miRNA-mimic on *firefly/renilla per se* and for validation of the particular target prediction. Dark grey columns show normalized *firefly* activities from pmirGLO (co)-transfections, light grey columns those from pmirGLO/CDK6-3^′^-UTRs (co)-transfections. Percent reductions of *firefly* activities of pmirGLO/CDK6-3^′^-UTRs compared to pmirGLO are given as well as their statistical significancies (at least two biological replicates with four technical replicates each, Student’s *t*-test, unpaired, ** p ≤ 0.01, ***: p ≤ 0.001). Summarizing results from both CDK6-3^′^-UTR fragments, significant regulatory miRNA effects were seen for miR-22, miR-26a, miR-26b, and miR-29b, whereas miR-137 had no significant effect.

Pairs of pmirGLO and pmirGLO/3^′^-UTR were cotransfected into HEK293T cells with the appropriate miRNA-mimic (Dharmacon) to assess the activity of a specific miRNA on a given 3^′^-UTR. Figures 2A and 2B depict *renilla*-normalized *firefly* activities measured from co-transfections of each mimic miR-10a, -22, -26a, -26b, -29b, and −137 with pmirGLO compared to co-transfections of these mimics with pmirGLO/CDK6-1-3^′^-UTR (Figure 2A) and pmirGLO/CDK6-2-3^′^-UTR (Figure 2B) respectively. The percentage reduction of relative firefly activities in pmirGLO/3^′^-UTR + mimic compared to activities of pmirGLO + mimic transfections correlate with miRNA activity on the given 3^′^-UTR. Here we identified strong interactions between CDK6-2 and miR-26a and miR-26b and moderate interactions with miR-29b. miR-22 and miR-29b interacted with fragment CDK6-1.

**Figure 3:**
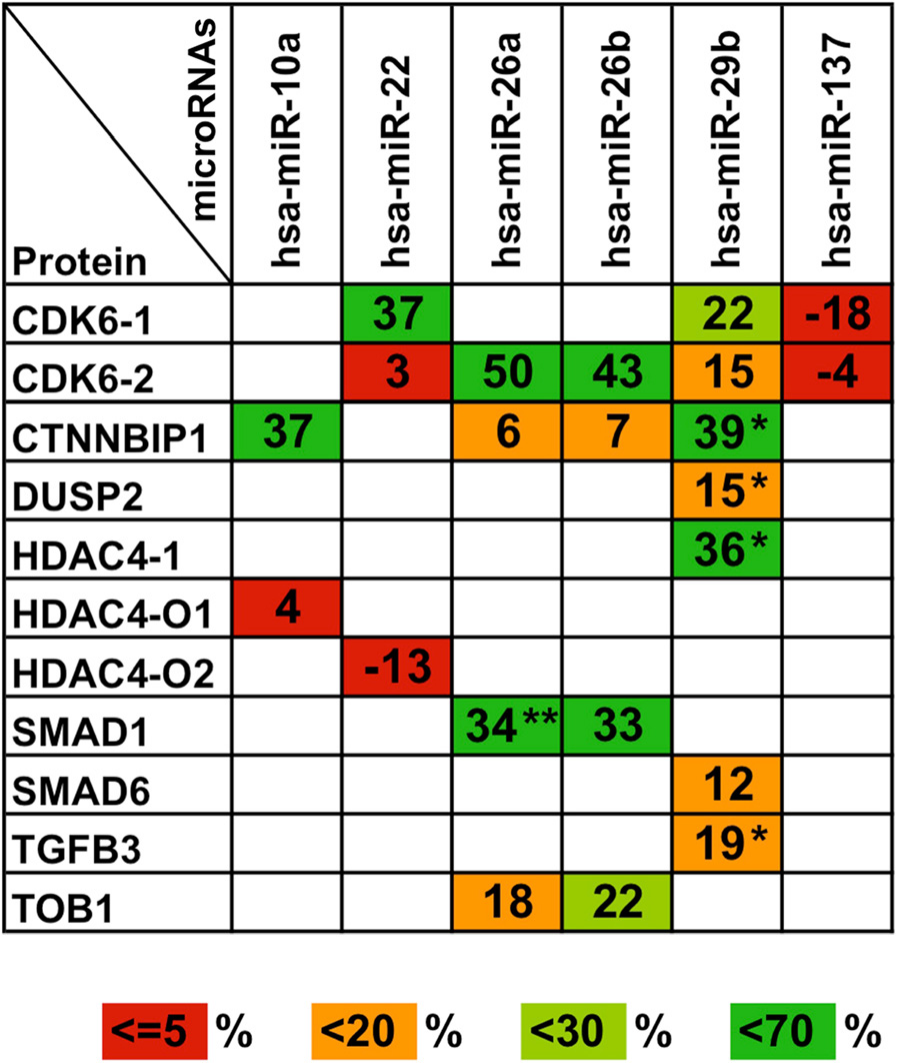
**Summary of miRNA-target gene validations.** In HEK293T-cells we tested the effects of a total of six miRNA-mimics (miR-10a, -22, -26a, 26b, 29b, and -137) on the 3^′^-UTRs of 8 predicted proteins (CDK6, CTNNBIP1, DUSP2, HDAC4, SMAD1, SMAD6, TGFB3, and TOB1) related to osteogenic differentiation and/or function. Percent reductions of normalized *firefly*-activities from pmirGLO/3^′^-UTR/miRNA-mimic co-transfections compared to pmirGLO/miRNA-mimic co-transfections are given in the colored squares. Red squares: lacking or non-significant miRNA effects, orange: weak but significant (p ≤0,05) miRNA effects, light green: moderate microRNA effects, dark green: strong microRNA effects. Among 22 individual miRNA-3^′^-UTR interactions analyzed, 10 were counted as positive (light and dark green squares). *: predictions already validated in [32], **: predictions already validated in [31]. These were analyzed again here for reasons of comparability to our other validations. Detailed data from all experimental validations are listed in Additional file 2.

Figure 3 summarizes the results of experimental validations from all 22 predicted miRNA-target interactions: CDK6 was targeted by miR-22, miR-26a, miR-26b, and miR-29b; CTNNBIP1 was regulated by miR-10a and miR-29b; SMAD1 and TOB1 were both recognized by miR-26a and miR-26b; and HDAC4 was targeted by miR-29b. Validations for DUSP2, SMAD6 and TGFB3 failed to give strongly positive results, with TGFB3 only weakly affected by miR-29b. Detailed data for all experimental validation studies are presented in Additional file 2. In summary, we identified osteo-inhibitory targets for miR-10a, miR-22, miR-26a, miR-26b, and miR-29b with the highest targeting impact resulting from miR-26a, miR-26b, and miR-29b expression.

### Target gene expression analysis and impact of regulating microRNAs

**Figure 4:**
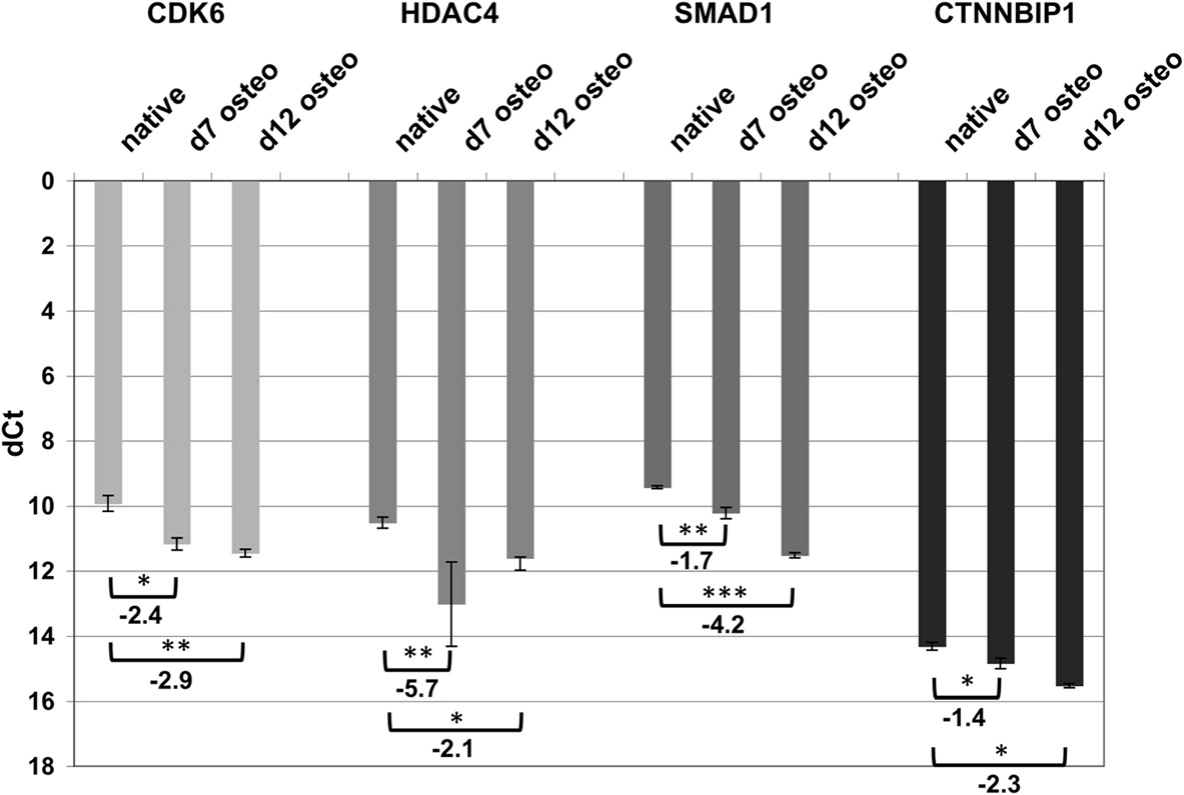
**qPCR analysis of expression profiles of CDK6, HDAC4, SMAD1, and CTTNBIP1 during osteogenic differentiation of USSC 86b at days 0 (native), 7, and 12.** ß-actin normalized dCt values as means from 4 replicates are shown, together with standard deviation and statistical significancies (Student’s *t*-test, unpaired, *: p ≤ 0.05, **: p ≤ 0.01, ***: p ≤ 0.001). Increased dCt values correspond to downregulation of transcripts during osteogenic differentiation and fold changes (2^–ddCt^) in expression between native and differentiated cells are given for each transcript.

As the availability of USSC lines SA5/73 and SA8/25 became limited in the course of our study, we focused on the newer USSC lines 86b and 77 for target gene expression analyses. Using qPCR, we analyzed transcript expression of validated targets CDK6, HDAC4, CTNNBIP1, SMAD1, and TOB1 during osteogenic differentiation of USSC line 86b at time points day 0 (native cells), and days 7 and 12 post DAG induction. Figure 4 demonstrates that CDK6, HDAC4, SMAD1, and CTNNBIP1 were all steadily downregulated at days 7 and 12 compared to day 0. HDAC4 was downregulated at day 7, followed by a slight increase in expression at day 12, albeit weaker than at day 0. We were unable to identify TOB1 transcripts in USSC. These results indicate inverse transcriptional regulation of miRNAs and target-mRNAs inhibiting osteogenic differentiation of USSC.

As miRNAs downregulate their targets on a posttranscriptional level, we also analyzed HDAC4, CDK6, and SMAD1 protein expression during osteogenic differentiation (up to day 12) of USSC 86b and in response to ectopic expression of miR-26a, miR-26b, and miR-29b in native USSC86b.

**Figure 5:**
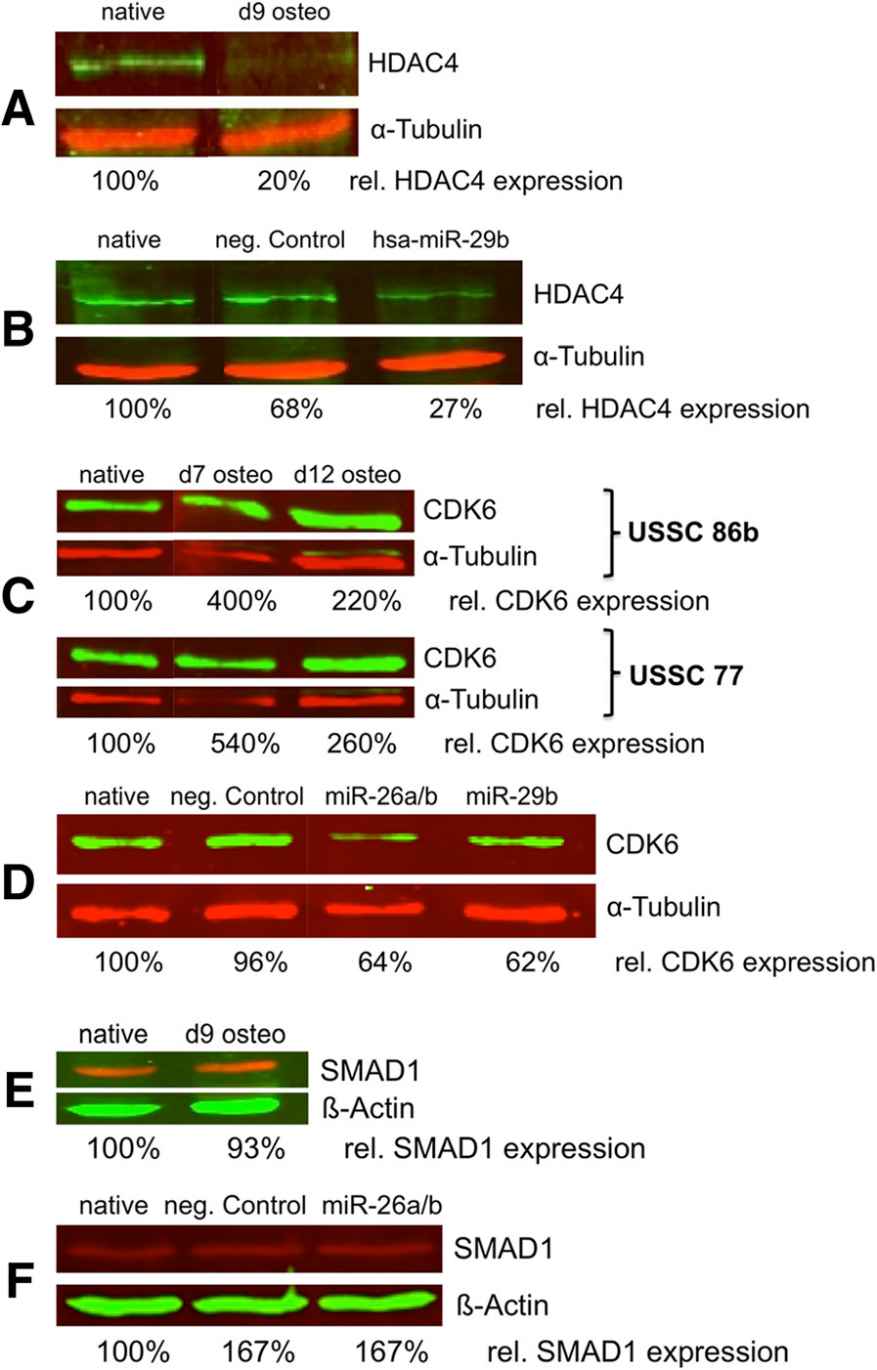
**Expression of HDAC4, CDK6, and SMAD1 in native and osteo-differentiated USSC as well as in response to regulating miRNAs.** Western-blots were scanned and protein expression levels quantified, normalized against the housekeeping proteins α-tubulin or ß-actin, and compared to expression in native/untransfected cells (percent values shown below the blot scans). (**A**) HDAC4 expression in native and osteo-differentiated (day 9) USSC 86b, (**B**) HDAC4 expression in USSC86b in response to transfection of a miR-29b mimic compared to transfection of a negative control small RNA and untransfected (native) cells. (**C**) CDK6 expression in native USSC 86b and USSC 77, on days 7 and 12 post DAG induction. (**D**) CDK6 expression in USSC 86b in response to transfection with miR-26a/b, miR-29b and a negative control small RNA as compared to native cells. (**E**) SMAD1 expression in native and day 9-osteo-differentiated cells USSC 86b, (**F**) SMAD1 expression in response to transfection of USSC 86b with miR-26a/b as compared to negative control transfected and native cells.

As seen in Figure 5A, HDAC4 was weakly expressed in native USSC 86b and downregulated by a factor 5 in day 9 osteo-differentiated USSC 86b, thereby matching our qPCR results (Figure 4). The HDAC4 protein level was also reduced following transfection with miR-29b (Figure 5B).

CDK6 behaved differently on the protein level; this protein was initially upregulated at day 7 of osteogenic differentiation in USSC 86b, and slightly downregulated at day 12 although still more abundant than in native cells (Figure 5C). To further examine this unexpected result, we tested an additional USSC line (USSC 77), which yielded the same dynamic CDK6 expression pattern (Figure 5C). Upon independent transfection with (i) an equimolar mixture of miR-26a and miR-26b mimics (both miRNAs share their seed sequence and differ in only two nucleotides) and (ii) with miR-29b mimics, CDK6 protein abundance was reduced compared to native and negative control cells 48h after transfection (Figure 5D). As with HDAC4, our results confirm that miR-26a, miR-26b, and miR-29b target CDK6.

Interestingly, SMAD1 expression remained unchanged at day 9 post DAG induction compared to native USSC 86b (Figure 5E) and was not affected by transfection with miR-26a and miR-26b mimics (Figure 5F). We were unable to detect the weakly transcribed CTNNBIP1 with any of several established antibodies (data not shown).

### Functional impact of miR-26a/b and miR-29b on osteogenic differentiation of USSC

Our experimental target validations indicate that miR-26a, miR-26b, and miR-29b likely have the strongest impact on osteogenic differentiation of USSC by reducing osteo-inhibitory CDK6 and HDAC4 proteins. We thus tested whether overexpression of miR-26a/b and miR-29b using miRNA mimics influences DAG-induced osteogenic differentiation. USSC SA5/73 and USSC 86b were each transfected with (i) a small unspecific negative control RNA, (ii) an equimolar batch of miR-26a and miR-26b, (iii) miR-29b, and (iv) an equimolar batch of miR-26a, miR-26b, and miR-29b mimics (SA5/73 only), each followed by DAG induction. Osteogenic differentiation was assessed by alizarin-red staining and calcium release at day 7 (both USSC lines).

**Figure 6:**
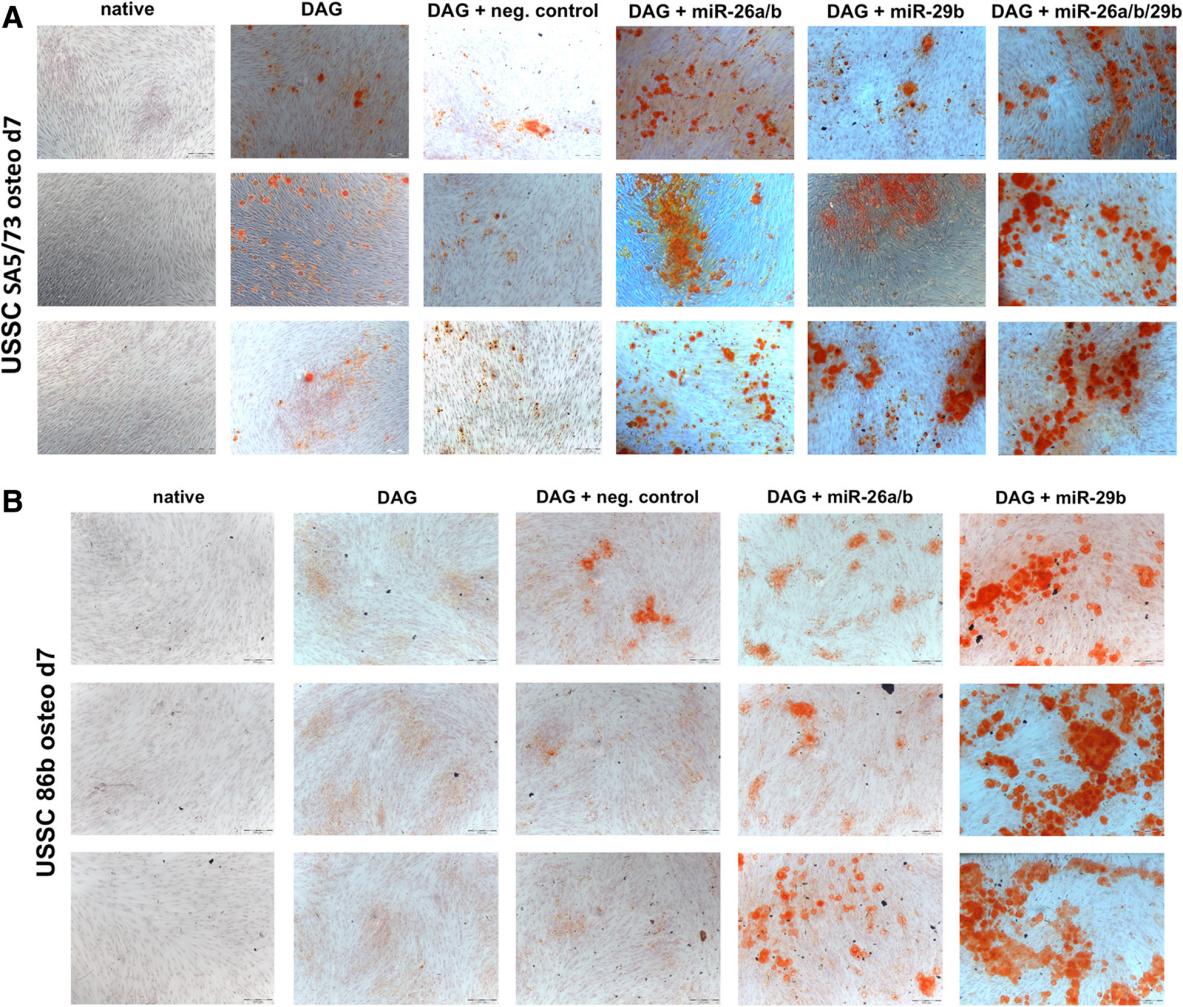
**Functional impact of miR-26a/b and miR-29b on osteogenic differentiation of USSC.** Impact of miR-26a/b and miR-29b on osteogenic differentiation on (**A**, scale bars 200μm) USSC SA5/73 at day 7 and (**B**, scale bars 500μm) on osteogenic differentiation of USSC 86b at day 7 post induction of DAG-induced differentiation was analyzed. Alizarin-red staining of native uninduced USSC and DAG-induced cells are shown in comparison to DAG-induced cells transfected with a negative control smallRNA, miRNA mimics miR-26a/b (equimolar batch), miR-29b, and miR-26a/b/29b (equimolar batch).

As seen in Figures 6A and 6B, both USSC lines started to differentiate at day 7 post DAG induction. Transfection of negative control RNA did not substantially influence alizarin-red staining in both USSC SA5/73 (Figure 6A) and USSC 86b (Figure 6B). In contrast, miR-26a/b-mimic transfected cells of both USSC lines showed significantly increased staining (Figures 6A and 6B). Transfection with miR-29b-mimic also resulted in accelerated osteogenic differentiation of both lines (Figures 6A and 6B). Transfection of USSC SA5/73 with miR-26a/miR-26b/miR-29b mimics further increased differentiation (Figure 6A).

**Figure 7:**
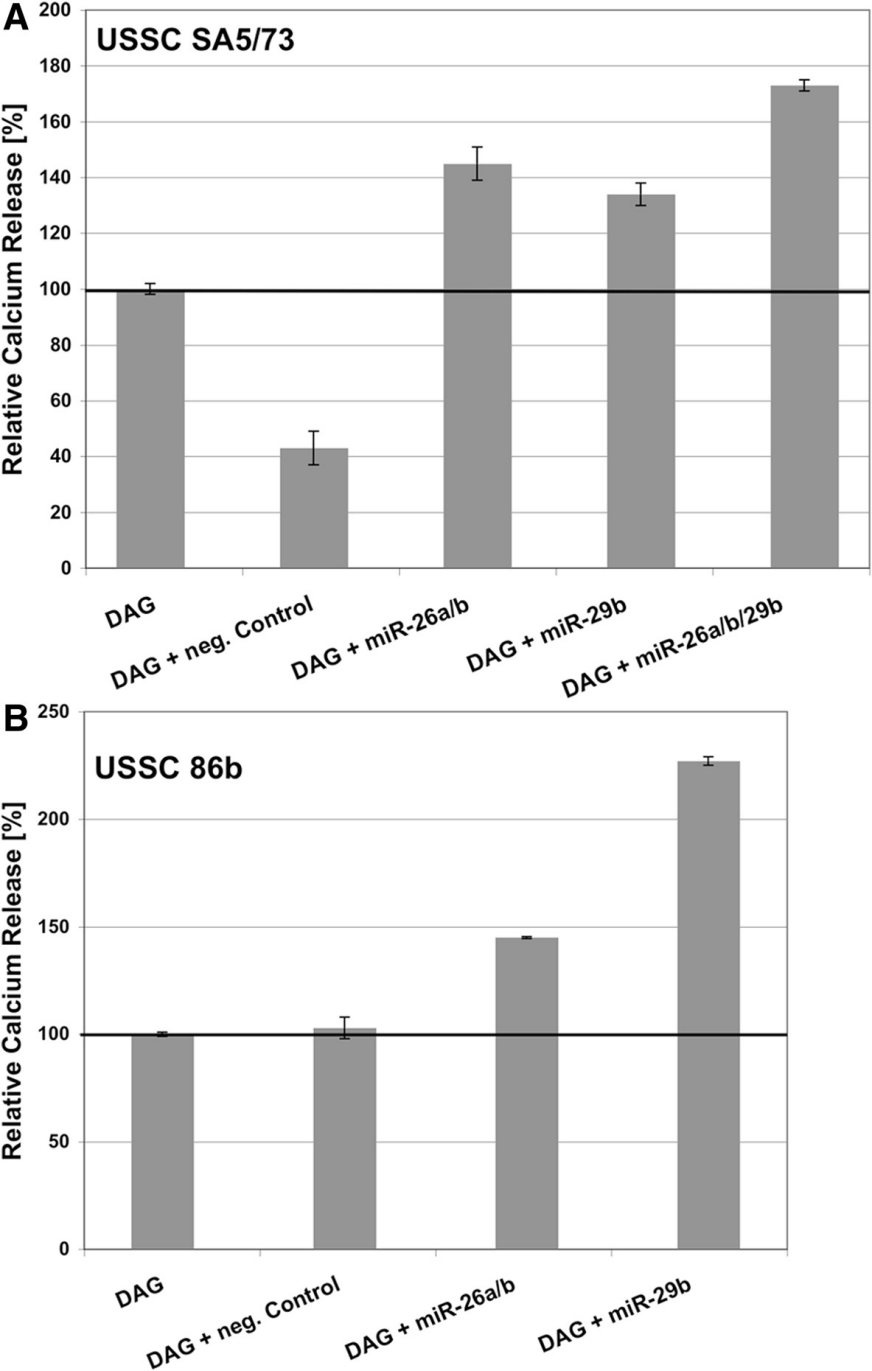
**Calcium-release assay from cells used in functional assays described in Figure**5**.** (**A**) USSC SA5/73, (**B**) USSC 86b. Percent mean values from experimental duplicates are given. In both cell lines, calcium release increased upon transfection with miR-26a/b and miR-29b followed by DAG-induction, as compared with untransfected/neg. control-transfected cells.

The finding that miR-26a, miR-26b, and miR-29b accelerated osteogenic differentiation of USSC was further supported by calcium release assays performed in DAG-induced USSC SA5/73 and 86b at days 0 and 7 of osteogenic differentiation. As seen in Figures 7A and 7B, calcium release increased upon transfection with miR-26a/b and miR-29b as compared to negative-control transfected and untransfected USSC SA5/73 (Figure 7A) and USSC 86b (Figure 7B). Transfection of SA5/73 with the miR-26a/miR-26b/miR-29b batch showed even higher calcium release, compared to miR-26a/miR-26b and miR-29b transfections alone (Figure 7A).

## Discussion

USSC are primary cells from human cord blood and are available in limited amounts from different patient sources. In general, USSC cell lines, such as SA5/73, SA8/25, 86b, and 77, are capable of comparable osteogenic differentiation as assessed by alizarin-red staining (data not shown). During osteogenic differentiation, only 30 miRNAs were commonly upregulated in two USSC lines (SA5/73 and SA8/25), although 124 and 196 miRNAs were respectively upregulated in each cell line. However, as expected for target-redundant miRNA networks, evaluation of commonly upregulated miRNAs revealed multiple bioinformatically osteo-inhibitory target genes.

miRNA expression profiling followed by target validation indicated that miR-26a, miR-26b, and miR-29b had the highest impact on osteogenic differentiation in our USSC lines. In the mouse osteoblast model, miR-29b is a positive regulator of osteogenic differentiation, able to increase differentiation on ectopic expression, with HDAC4, TGFB3, ACVR2A, CTNNBIP1 and DUSP2 as validated targets [32]. Here we show a comparable function for miR-29b in osteogenic differentiation of human somatic stem cells confirming human CTNNBIP1 and HDAC4 as miR-29b targets in our HEK293T-cell based validation assay. CTNNBIP1 was also regulated by miR-10a and CDK6 [45] was targeted by miR-22, miR-26a, miR-26b and miR-29b.

miR-26a modulates late osteogenic differentiation of hADSC through SMAD1 targeting [31] and we showed that both, miR-26a and miR-26b regulate SMAD1, this protein is known as a positive mediator of osteogenic differentiation [59]. We also identified the osteo-inhibitory BMP/SMAD regulator TOB1 [51] as a target of miR-26a and miR-26b. Substantial homology between these two miRNAs (identical seed sequences) is reflected in their similar behavior in our target validation assays. To clarify the contradictory roles of osteo-promoting SMAD1 and osteo-inhibitory CDK6 and TOB1 as parallel targets of miR-26a and miR-26b, we directly analyzed target protein abundance by quantitative Western blotting (i) during osteogenic differentiation of USSC and (ii) in response to transfection of USSC with miRNA mimics.

Despite targeting of the SMAD1 3^′^-UTR by miR-26a and miR-26b in our luciferase assay (Figure 3), SMAD1 protein abundance remained unaltered upon transfection with miR-26a/b mimics (Figure 5F). This finding indicates either a long intracellular half-life for SMAD1 in USSC, unaltered by miRNA transfection in the experimental timeframe, or secondary regulatory mechanisms such as increased transcription that keep SMAD1 levels constant. Accordingly, SMAD1 protein levels also remained constant at day 9 in osteo-differentiated USSC 86b (Figure 5E) as expected for an osteo-promoting protein. However, our qPCR data indicate that SMAD1 transcription decreased steadily from day 0 to day 7 and to day 12 of osteogenic differentiation (Figure 4), further supporting a long half-life for SMAD1 protein. Due to strong calcification of USSC at day 12, we were unable to measure SMAD1 protein and it remains unclear whether this protein has a long half-life or is regulated by other unidentified mechanisms.

In contrast to osteo-promoting SMAD1, osteo-inhibitory CDK6 protein expression was indeed reduced 48h post transfection with miR-26a, miR-26b, and miR-29b mimics. This finding indicates a comparatively strong regulatory influence of miR-26a/b and miR-29b on CDK6. However, despite steady transcript downregulation, CDK6 protein expression peaked at day 7 of osteogenic differentiation, before decreasing. As miRNAs act postranscriptionally [4,60], the observed decrease of CDK6 abundance at day 12 was likely due to continued upregulation of miRNAs targeting osteo-inhibitory factors beyond day 7, after achieving sufficient levels to alter CDK6 protein abundance.

The HDAC4 gene product was reduced on both transcript (Figure 4) and protein level (Figure 5B) during osteogenic differentiation. Similarly, the HDAC4 protein level was reduced upon transfection with miR-29b mimic, consistent with our target validation assays. It should be noted that we were unable to detect either TOB1 transcripts or TOB1 and CTNNBIP1 gene products in USSC (data not shown).

Using alizarin-red staining and calcium release assays, we clearly demonstrated that miR-26a/b and miR-29b mimic transfections specifically accelerate osteogenic differentiation in both USSC lines tested. This result is consistent with the observation that miR-29b contributes to osteogenic differentiation of mouse osteoblasts [32]. It should be noted that attempts to functionally analyze miR-10a and miR-22 failed due to a nearly complete loss of transfected USSC from the culture plates (data not shown).

Since miR-26a/b and miR-29b regulate osteo-inhibitory and osteo-promoting factors in parallel, the osteo-inhibitory effects of CDK6 and HDAC4 likely outweigh the osteo-promoting effects of SMAD1; this finding is further supported by the unaltered abundance of SMAD1 in miR-26a/b transfected USSC.

The strongest effect on osteogenic differentiation was observed by transfecting an equimolar mixture of miR-26a, miR-26b, and miR-29b mimics. It is likely that miR-26a/b and miR-29b influence a common set of target genes with each miRNA making additional contributions through targeting exclusive genes e.g. HDAC4 and CTNNBIP1, which are regulated by miR-29b but not by miR-26a/b.

Recently miR-135b was reported to be 100-fold upregulated in USSC following DAG-induction [61]. In contrast, Li and coworkers reported that miR-135b (and also miR-133a/b) were downregulated during mouse osteoblast differentiation after 16 hours [30]. In USSC, miR-133a and miR-133b as well as miR-135b are only weakly expressed even in native cells and virtually unchanged during osteogenic differentiation (see Additional file 1).

A recent study of miRNA expression signatures asssociated with osteogenic commitment of USSC showed upregulation of similar miRNAs (miR-26b, miR-30b, let-7a, let-7f and miR-181a) as determined in our analysis [62]. However the authors used USSC from a different source, employed different miRNA expression analysis methodology (hybridisation array), and focused on other miRNAs, precluding direct comparison.

Taken together, our results further establish the importance of miRNAs in differentiation processes of USSC. We clearly demonstrated the combined functional impact of miR-26a/b and miR-29b, which had individually been identified as modulators of osteogenic differentiation in hADSC [31] and mouse osteoblasts [32]. Target gene similarities and differences between these miRNAs imply that these miRNAs act in a synergistic manner to improve and accelerate osteogenic differentiation of USSC.

## Conclusions

In summary, we detected a subset of miRNAs, notably miR-26a, miR-26b and miR-29b, which is consistently upregulated during osteogenic differentiation of USSC. We experimentally identified specific osteo-inhibitory proteins as regulatory targets for these miRNAs in reporter gene analyses and in direct measurements of target protein abundance. Functional analyses demonstrated that miR-26a, miR-26b and miR-29b positively modulate osteogenic differentiation of USSC, most likely by downregulating osteo-inhibitory target proteins. Together with our previous studies on neuronal lineage differentiation [38], [39] these findings further support the notion that differentiation of the unique somatic stem cell type USSC follows established biochemical pathways wherein miRNAs are important regulatory molecules.

## Methods

### Generation and osteogenic lineage differentiation of USSC

USSC lines were isolated from human cord blood and characterized in a designated unit in our institute under GMP conditions as described in detail in [33] and [63] and were supplied to us for this study. Informed consent was obtained from each participant.

USSC lines SA5/73 and SA8/25 were induced to osteogenic differentiation on addition of DAG (dexamethasone, ascorbic acid, ß-glycerolphosphate). Cells were incubated for 7 days and osteogenic differentiation was assessed using Alizarin-Red staining as described [33]. Calcium release was measured following incubation of cells in 6M HCl for 24h at 37°C; calcium content in the supernatant was analyzed using the Calcium Colorimetric Assay (BioVision) according to the manufacturer’s instructions followed by normalization to the protein content of the sample.

### miRNA expression analysis

Small and large RNA fractions were isolated from native and differentiated USSC lines SA5/73 and SA8/25 using the Ambion *mir*Vana miRNA Isolation kit (Applied Biosystems, Darmstadt, FRG) according to the manufacturer’s instructions; adherent USSC were directly lysed without cell trypsinization. Total RNA for small RNA sequencing was prepared using TRIzol reagent (Invitrogen, FRG).

miRNA expression analyses were performed on small RNA fractions using the TaqMan miRNA Megaplex array (pool A, Applied Biosystems, Darmstadt, FRG) [43] according to the manufacturer’s instructions. Briefly, 10ng small RNA fraction was reverse transcribed and preamplified for 12 PCR cycles, with subsequent TaqMan-probe based array-amplification for 40 additional PCR cycles. Raw Ct-values were normalized to U6 RNA data and ddCt as well as 2^-(ddCt)^ data were calculated.

Barcoded small RNA sequencing was used to generate miRNA expression profiles for four native USSC lines (SA5/73, SA8/25, SA8/77, SA4/101) and two day 7-osteo-differentiated USSC lines (SA8/77 and SA4/101); this method is a modification of an established small RNA sequencing protocol which involves sequential ligation of 3^′^ and 5^′^ adapters to small RNAs, followed by cDNA library preparation, Solexa sequencing, and small RNA annotation [56].

### Bioinformatic target gene predictions

Most miRNA targets were predicted using the miRGen engine (http://www.diana.pcbi.upenn.edu/miRGen.html); additional targets were identified using miRanda (http://www.microrna.org/microrna/getGeneForm.do), PicTar (http://pictar.mdc-berlin.de/), and TargetScan webtools (http://www.targetscan.org/). Further analyses (*gene ontology*) of predicted targets were performed using the DAVID database (http://david.abcc.ncifcrf.gov/home.jsp).

### Experimental verification of bioinformatic target gene predictions

PCR-products of full length or fragments of 3^′^-UTRs as well as double-stranded oligonucleotides covering the predicted miRNA binding sites on the target mRNA of interest were cloned at the 3^′^-end of *firefly* luciferase ORF in dual reporter (*firefly* and *renilla* luciferases) vector pmirGLO (GenBank accession FJ376737, Promega Mannheim, FRG) using restriction enzyme pairs SacI/XbaI or SalI/XhoI. PCR primers, sense and antisense oligonucleotides, and 3^′^-UTR- and fragment-lengths are listed in Additional file 3. To normalize for effects of endogenous miRNAs on a given 3^′^-UTR, 100ng pmirGLO and pmirGLO/3^′^-UTR were transfected into 5×10^4^ HEK293T cells using 0.5 μl Lipofectamine 2000 (Invitrogen, Karlsruhe, FRG). Pairwise cotransfections of 100ng empty pmirGLO with the 2.5 pmol miRNA mimic (Dharmacon, Bonn, FRG) of interest and pmirGLO/3^′^-UTR with the miRNA mimic of interest were performed to determine the influence of the given miRNA on the 3^′^-UTR. *Firefly* and *renilla* activities were measured 24h after transfection using Beetlejuice and Renillajuice reagents (PJK, Kleinblittersdorf, FRG). All transfection experiments were performed in at least two independent biological experiments with quadruple transfections each and statistical significances were calculated by a student’s t-test, unpaired.

### Real-time PCR analysis

Total RNA was isolated using Trizol reagent (Invitrogen) according to the manufacturer’s instructions. RNAs were reverse transcribed using M-MLV reverse transcriptase (Promega, Mannheim, FRG) and real-time PCRs were performed using the Maxima SYBR Green/ROX qPCR Master Mix (Fermentas, St. Leon-Rot, FRG). Primers for CDK6 [64], CTNNBIP1 [65], SMAD1 [31] and HDAC4 (this work) used in RT-reactions are listed in Additional file 3. Results were analyzed using Step One Software v2.1 and statistical significances were calculated by a student’s t-test, unpaired.

### Transfections of USSC for Western blots and functional analyses

To analyze changes in endogenous protein abundance during osteogenesis, USSC were cultivated on 6-well plates and induced with DAG as described above. Proteins were isolated at days 0 (native), 7, 9, and 12. To evaluate the impact of miRNAs on endogenous target proteins, USSC were cultivated on 6-well plates and transfected with 40pmol of relevant miRNA-mimic/well using Dharmafect1 reagent according to the manufacturer’s instructions, proteins were isolated 48h after transfection. Native, osteo-differentiated and transfected USSC were washed with PBS and lysed using RIPA-Buffer (Sigma, FRG) supplemented with protease inhibitor cocktail tablets (Roche, FRG). Up to 50μg of proteins/lane were separated by SDS-PAA electrophoresis, blotted onto nitrocellulose membranes (0.45μm), and membranes were blocked with 3% Milk/PBS, incubated with primary antibodies (see below) in 3% milk/PBS followed by incubation with the appropiate secondary antibody (see below) in 3% milk/PBS/0,15% Tween-20. Membranes were washed in PBS-T/0.1% Tween-20, dried, and scanned with the LI-COR Odyssey Infrared Imager (LI-COR Biosystems). Visible false-color signals were quantified using Odyssey 2.1 software (LI-COR Biosystems) and normalized against signals from quantified housekeeping proteins α-Tubulin or ß-Actin. The following antibodies were used in this study: CDK6, sc-177; HDAC4, sc-11418; SMAD1, sc-7965 (all Santa Cruz Biotechnology), α-Tubulin, B-5-1-2 (Sigma Aldrich); ß-Actin, ab34731 (abcam); IRDye 680LT α-mouse and IRDye 800CW α-rabbit (both: LI-COR Biosystems).

For functional analyses, USSC were cultivated on 24-well plates and transfected with 10pmol miRNA-mimic/well using Dharmafect1 reagent according to the manufacturer’s instructions. Transfected cells were induced to osteogenic differentiation 24 h post transfection as described above. Differentiation was analyzed at day 7 by alizarin-red stainig and calcium released as described above.

### Ethics statement

Work with USSC was approved by the Ethics Committee of the Medical Faculty, University Düsseldorf, study numbers 3436 and 2975.

## Competing interests

The authors declare that they have no competing interests.

## Authors' contributions

HIT conceived and supervised the study, participated in molecular biology and protein biology experiments and wrote the manuscript. JD performed all miRNA analyses including bioinformatics, target validations and functional experiments. EH carried out all Western blot experiments. KMI delivered RT-PCR based miRNA expression analyses, NR, MH, and TT delivered deep sequencing data, and PW supervised the study. All authors read and approved the final manuscript.

## Supplementary Material

Additional file 1 **Comprehensive miRNA expression data from native (d0) USSC SA5/73 and SA8/25 and osteo-differentiated (day 7) SA5/73 and SA8/25.** This Excel file contains three separate sheets, “USSC SA573”, “USSC SA825”, and “Deep Sequencing”. In the first two, raw Ct, U6-RNA normalized dCt, ddCt, and 2^-ddCt^ values (corresponding to fold changes of miRNA expression in day 7 osteo vs. native cells, in grey columns) are given for each miRNA analyzed. The sheet “deep sequencing” indicates results from deep-sequencing derived expression analysis of miR-26a and miR-26b in native USSC SA5/73, SA8/25, SA8/77, and SA4/101 and osteo-differentiated USSC SA8/77 and SA4/101. Total sequence reads for the miRNA indicated as well as frequency of specific sequence reads among all sequence reads are given. Click here for file

Additional file 2 **Experimental validation of miRNA target gene predictions.** Detailed data of validations of CTNNBIP1 (A), DUSP2 (B), HDAC4-1 (C), HDAC4-O1 and HDAC4-O2 (D), SMAD1 (E), SMAD6 (F), TGFB3 (G), and TOB1 (H) are given. The bar above the graphs depict the respective 3^′^-UTRs, with the blue part representing the analyzed fragment, including putative miRNA binding sites. For further description see Figure 2. Click here for file

Additional file 3 **Primers and oligonucleotides.** This Excel file summarizes primers and sense- and antisense-oligonucleotides used for 3^′^-UTR cloning and qRT-PCR analysis. Sheet “3^′^UTR cloning”: Primers for generation of PCR products from 3^′^-UTRs, corresponding NCBI accession numbers, and sense- and antisense-oligonucleotides used for experimental target validation of predicted target proteins. Restriction sites used for cloning are given in bold as well as sticky and recessed ends synthesized into oligonucleotides. “Ⓟ” denotes the phosphate group added to the 5^′^-end of oligonucleotides. Mutations in oligonucleotide sequences corresponding to the seed of microRNAs are given in red. Sheet “qRT-PCR”: Forward and reverse primers used for RT-reaction. Click here for file
