# Supplementary material for: MicroRNAs miR-26a, miR-26b, and miR-29b accelerate osteogenic differentiation of unrestricted somatic stem cells from human cord blood
Source: BMC Genomics. 2013 Feb 19;14:111. doi: 10.1186/1471-2164-14-111 (PMC3637629; doi:10.1186/1471-2164-14-111)
Supplement: Additional file 2 — Experimental validation of miRNA target gene predictions. Detailed data of validations of CTNNBIP1 (A), DUSP2 (B), HDAC4-1 (C), HDAC4-O1 and HDAC4-O2 (D), SMAD1 (E), SMAD6 (F), TGFB3 (G), and TOB1 (H) are given. The bar above the graphs depict the respective 3′-UTRs, with the blue part representing the analyzed fragment, including putative miRNA binding sites. For further description see Figure 2. [file 1471-2164-14-111-S2.ppt]

## Slide 1
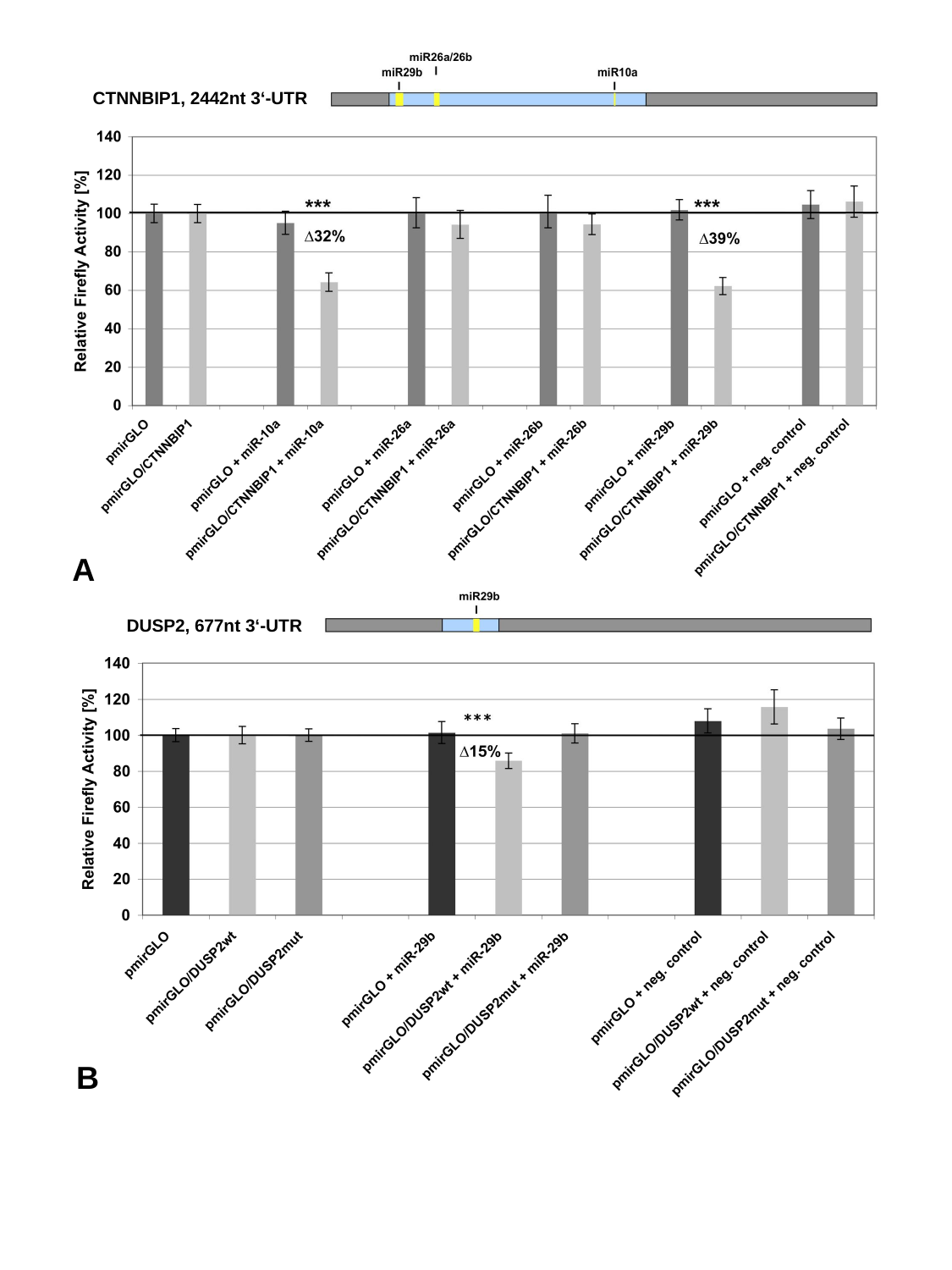

CTNNBIP1, 2442nt 3‘-UTR
A
DUSP2, 677nt 3‘-UTR
B

## Slide 2
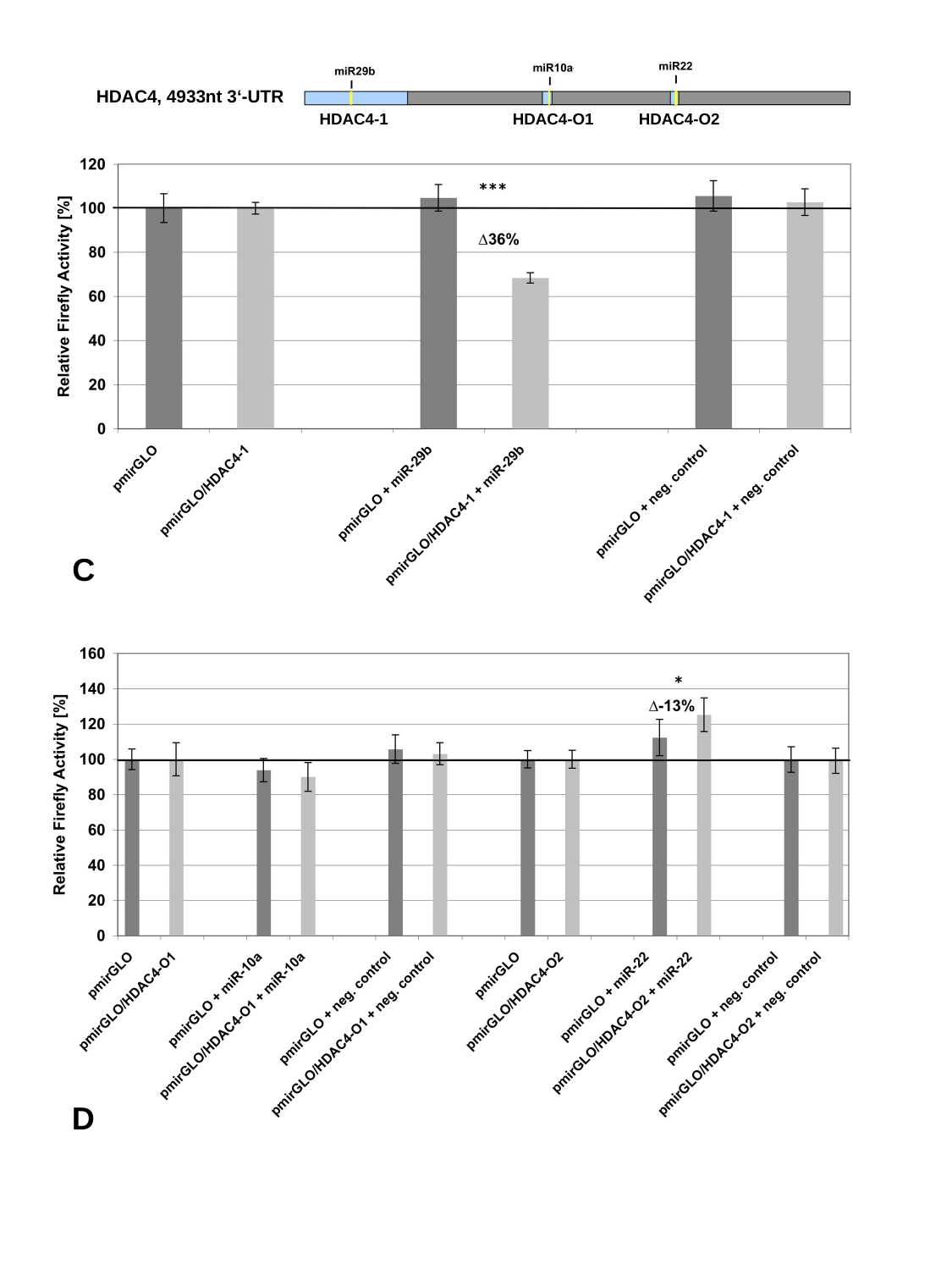

HDAC4, 4933nt 3‘-UTR
HDAC4-1
HDAC4-O1
HDAC4-O2
C
D

## Slide 3
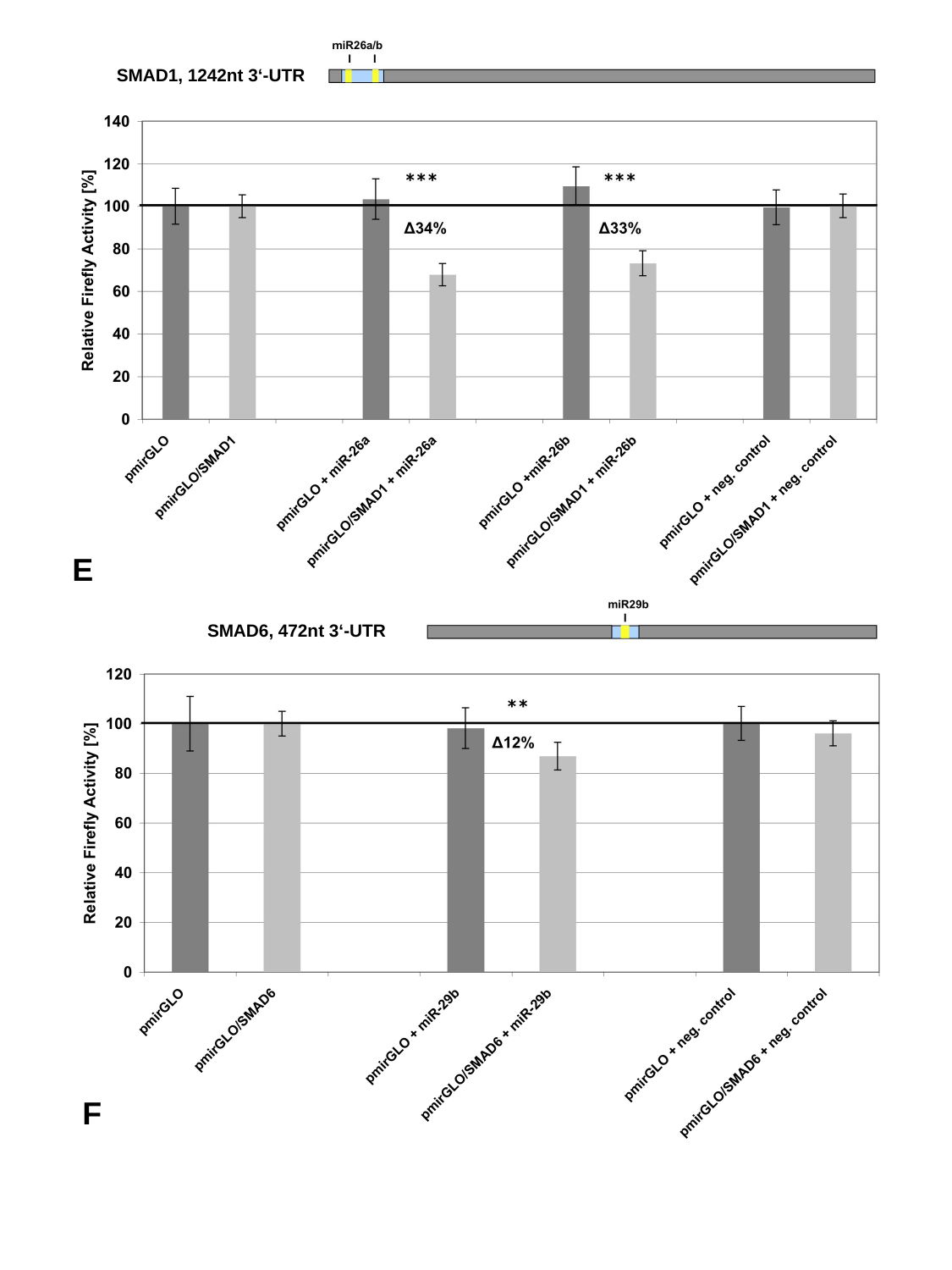

SMAD1, 1242nt 3‘-UTR
E
SMAD6, 472nt 3‘-UTR
F

## Slide 4
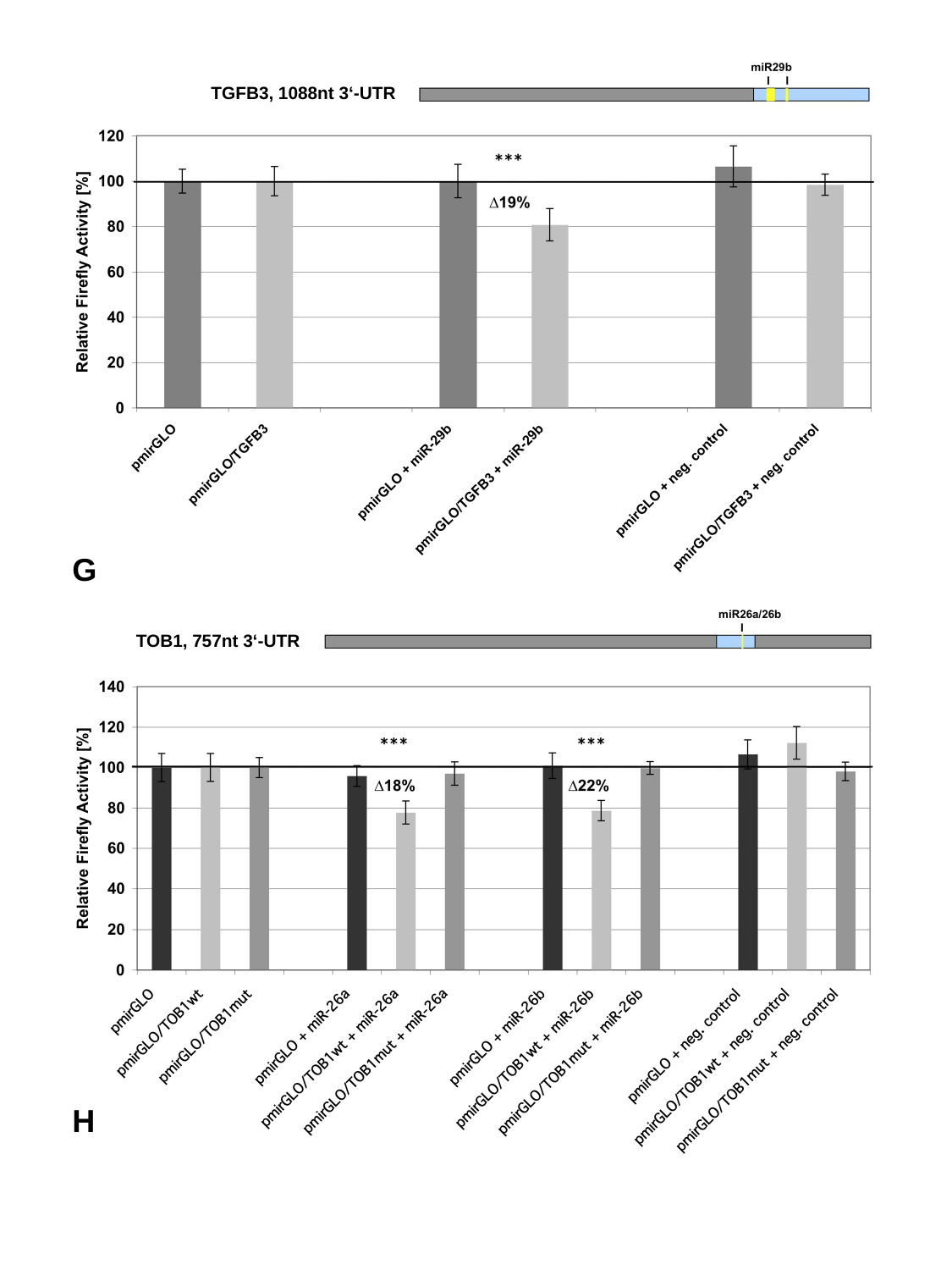

TGFB3, 1088nt 3‘-UTR
G
TOB1, 757nt 3‘-UTR
H
